# Supplementary material for: Maintenance of neurotransmitter identity by Hox proteins through a homeostatic mechanism
Source: Nat Commun. 2022 Oct 15;13:6097. doi: 10.1038/s41467-022-33781-0 (PMC9569373; doi:10.1038/s41467-022-33781-0)
Supplement: Supplementary file 1 — Supplementary Information [file 41467_2022_33781_MOESM1_ESM.pdf]

SUPPLEMENTARY INFORMATION

Supplementary Figures 1 – 8

Maintenance of neurotransmitter identity by Hox proteins through a homeostatic mechanism

Weidong Feng[1, 2, 3], Honorine Destain[1, 2, 3], Jayson J Smith[1, 2], Paschalis Kratsios[1,2,3]

[1] Department of Neurobiology, University of Chicago, Chicago, IL, USA.

[2] University of Chicago Neuroscience Institute, Chicago, IL, USA.

[3] Committee on Development, Regeneration, and Stem Cell Biology, University of Chicago, Chicago, IL, USA.

Correspondence: pkratsios@uchicago.edu

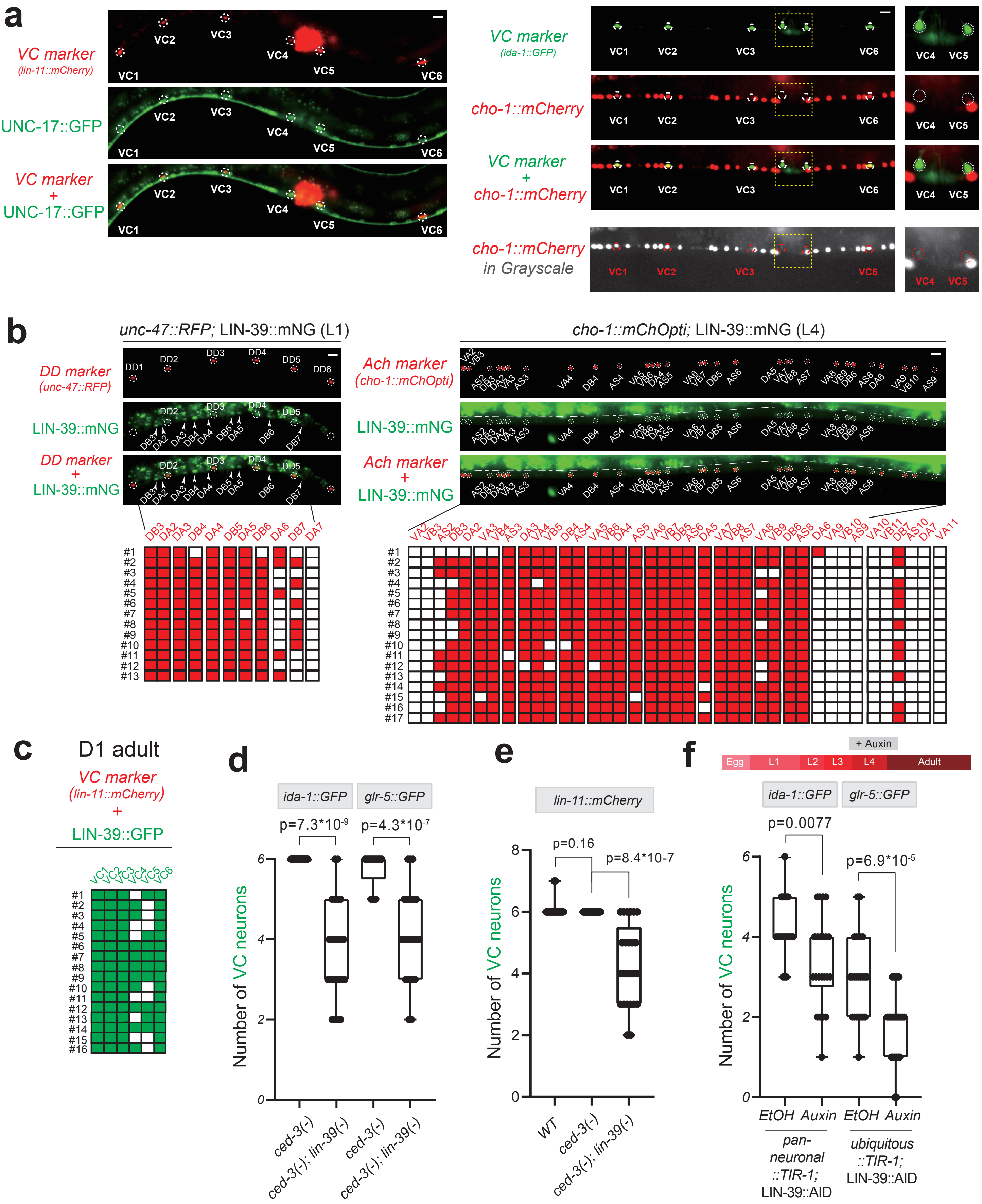

Supplementary Figure 1. LIN-39 is necessary to maintain VC terminal identity features.

a. Markers for *cho-1* and *unc-17* are expressed in all six VC MNs. Representative images showing *unc-17* (left) and *cho-1* (right) expression that colocalizes and known VC reporters in split and merged channels. Because *cho-1* expression is dim in VC4-5, a grayscale channel is shown **b-c**. LIN-39 is continuously expressed in MNs that control locomotion and egg-laying. Representative images of endogenous *lin-39* reporter (*lin-39::mNG*). Markers of GABAergic (*unc-47::RFP*) and cholinergic (*cho-1::mChOpti*) motor neurons were used to identify the *lin-39*-expressing neurons with single-cell resolution. For panel **c** refer to **Figure 1b** for representative image. **d-e**. Quantification of *ida-1, glr-5*, and *lin-11* reporter gene expression in day 1 adult animals of *ced-3 (-)* and *ced-3(-); lin-39(-)* genotypes. **f**. LIN-39 is required to maintain *ida-1* and *glr-5* expression in adult VC neurons. Timeline for auxin application indicated on top with quantifications below. Box and whisker plots were used with presentation of all data points. Box boundaries indicate the 25th and 75th percentile. The limits indicate minima and maxima values, whereas centre values (mean) are highlighted in red. Unpaired t-test (two-sided) with Welch's correction was performed and p-values were annotated. n = 17-24 animals. Source data are provided as a Source data file. Scale bars: 5 μm.

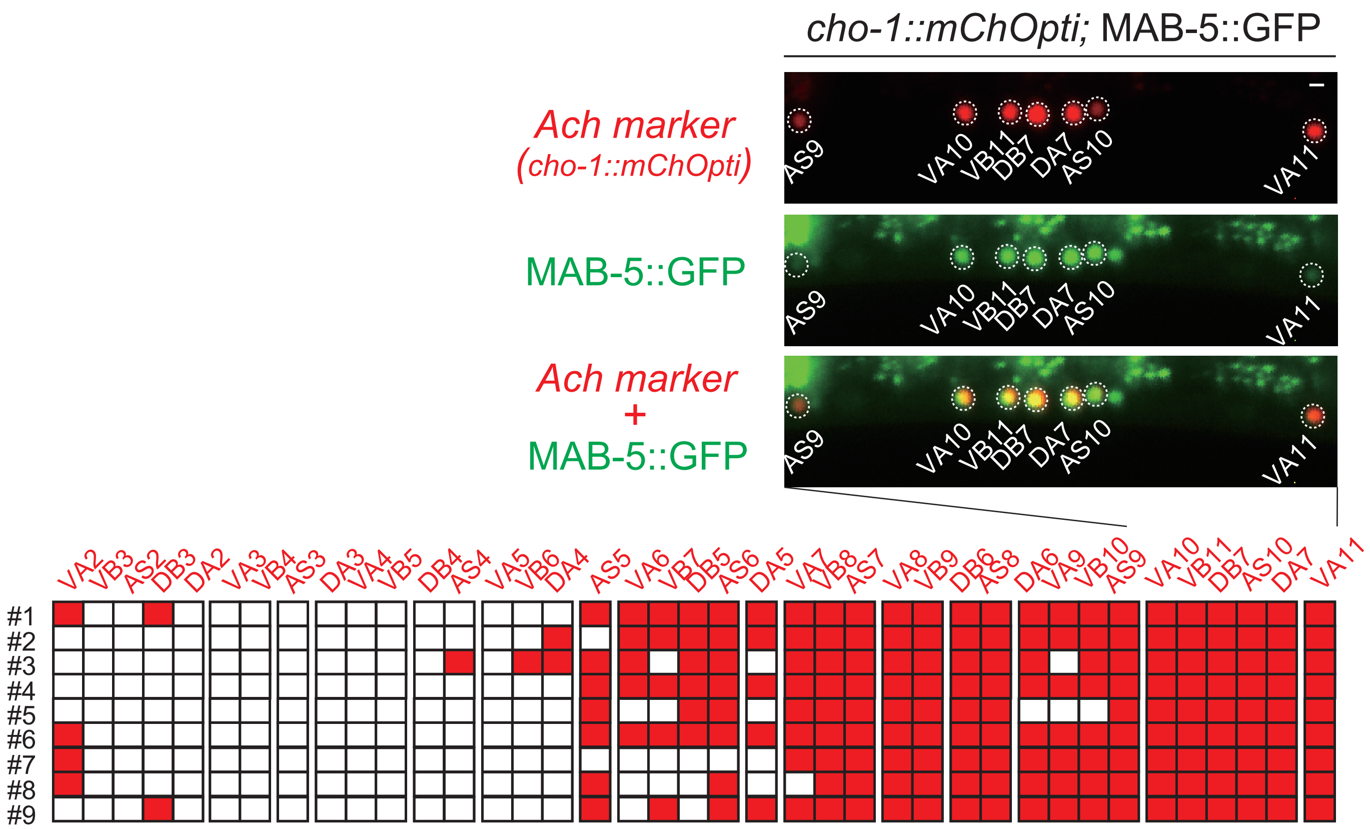

Supplementary Figure 2. MAB-5 expression profile in cholinergic MNs.
Representative co-localization images of MAB-5::GFP (fosmid reporter) with a cholinergic reporter that localizes to the nucleus (*cho-1::SL2::mChOpti::H2B*). Motor neuron nuclei are circled. L4 animals were analyzed for MN co-localization of the GFP and mChOpti markers and the results are shown in a grid format below. n = 9 animals. The experiment was repeated twice. Scale bar: 2 μm.

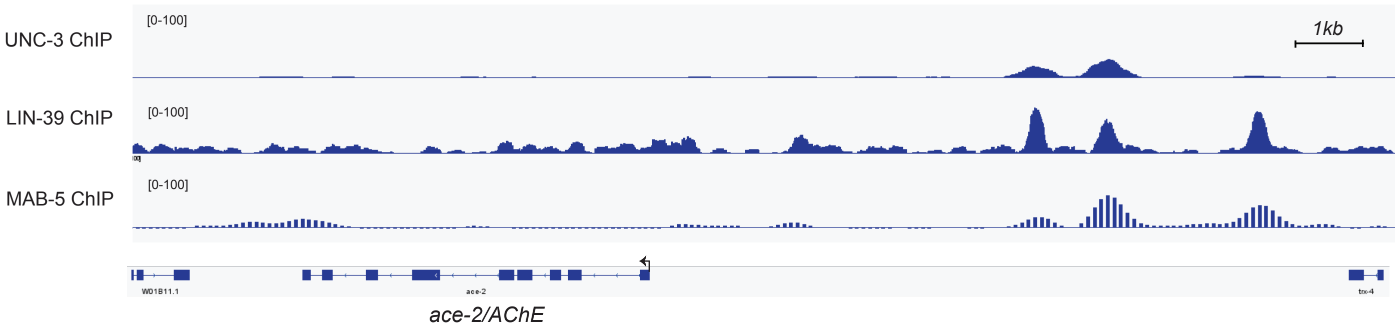

Supplementary Figure 3. ChIP-seq binding peaks for UNC-3, LIN-39 and MAB-5 on *ace-2* locus.
Schematic extracted from the IGV software showing the *ace-2* and ChIP-seq tracks for UNC-3, LIN-39 and MAB-5.

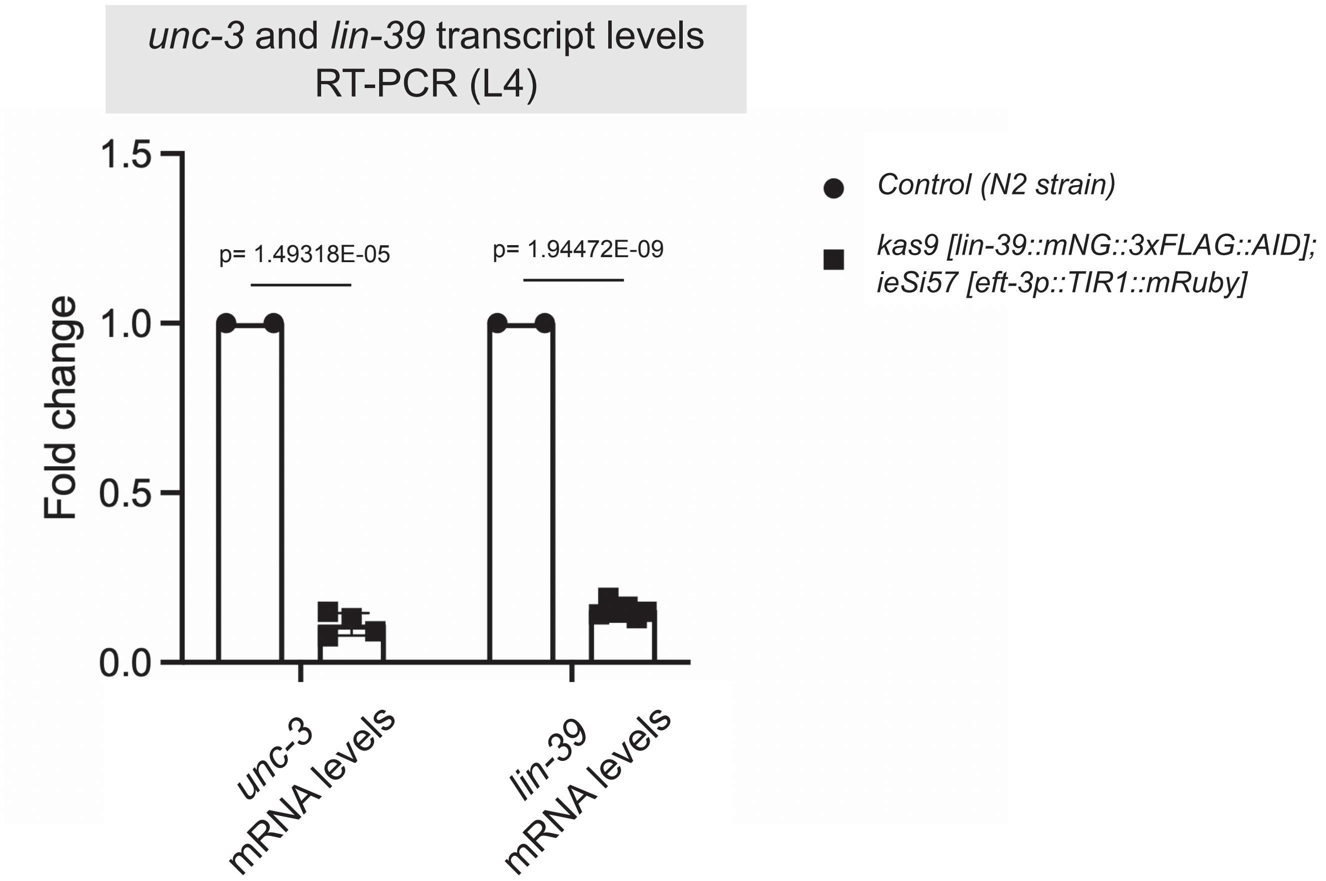

Supplementary Figure 4. Expression analysis of *unc-3* in animals carrying a hypomorphic *lin-39* allele. RT-PCR on whole worm lysates was used to measure *unc-3* and *lin-39* mRNA levels at synchronized animals (L4 stage). Compared to wild-type (N2 strain) animals, the levels of *lin-39* and *unc-3* transcripts are reduced in *lin-39 (kas9 [lin-39::mNG::3xFLAG::AID]); ieSi57[eft-3p::TIR1::mRuby]* animals. Data are presented as mean values with error bars indicating standard deviation (S.D.). Unpaired t-test (two-sided) with Welch's correction was performed and p-values are annotated. The experiment was repeated twice.

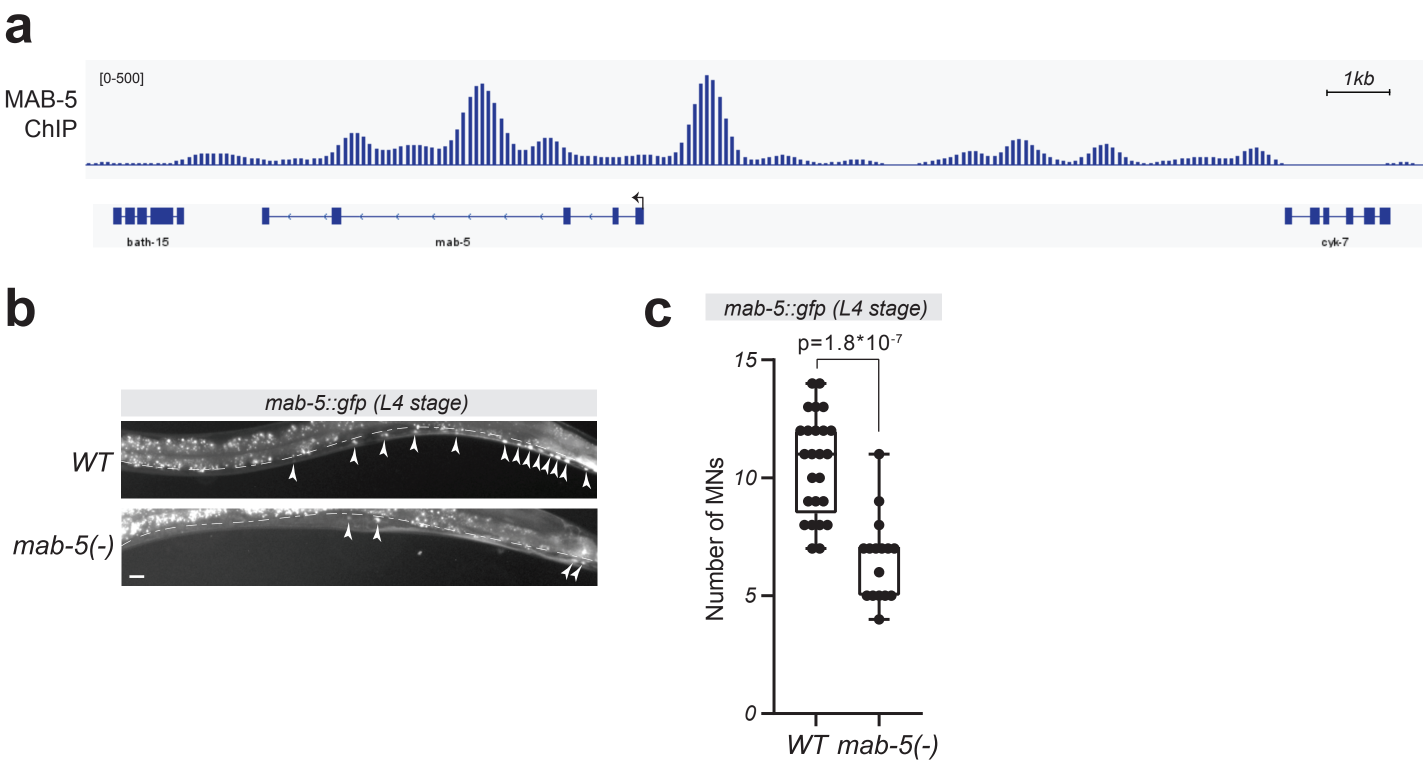

Supplementary Figure 5. Transcriptional autoregulation of *mab-5* in motor neurons.
a. Gene locus of *mab-5* with ChIP-seq tracks for MAB-5. **b**. Representative images of a *mab-5* transcriptional *gfp* reporter in posterior motor neurons of WT and *mab-5(e1239)* mutant animals at L4. **c**. Quantification of *mab-5::gfp* expression in panel **b**. Box and whisker plots were used with presentation of all data points. Box boundaries indicate the 25th and 75th percentile. The limits indicate minima and maxima values, whereas centre values (mean) are highlighted in red. Unpaired t-test (two-sided) with Welch's correction was performed and p-values were annotated. N = 16-25 animals. Source data are provided as a Source data file. Scale bar: 5 μm.

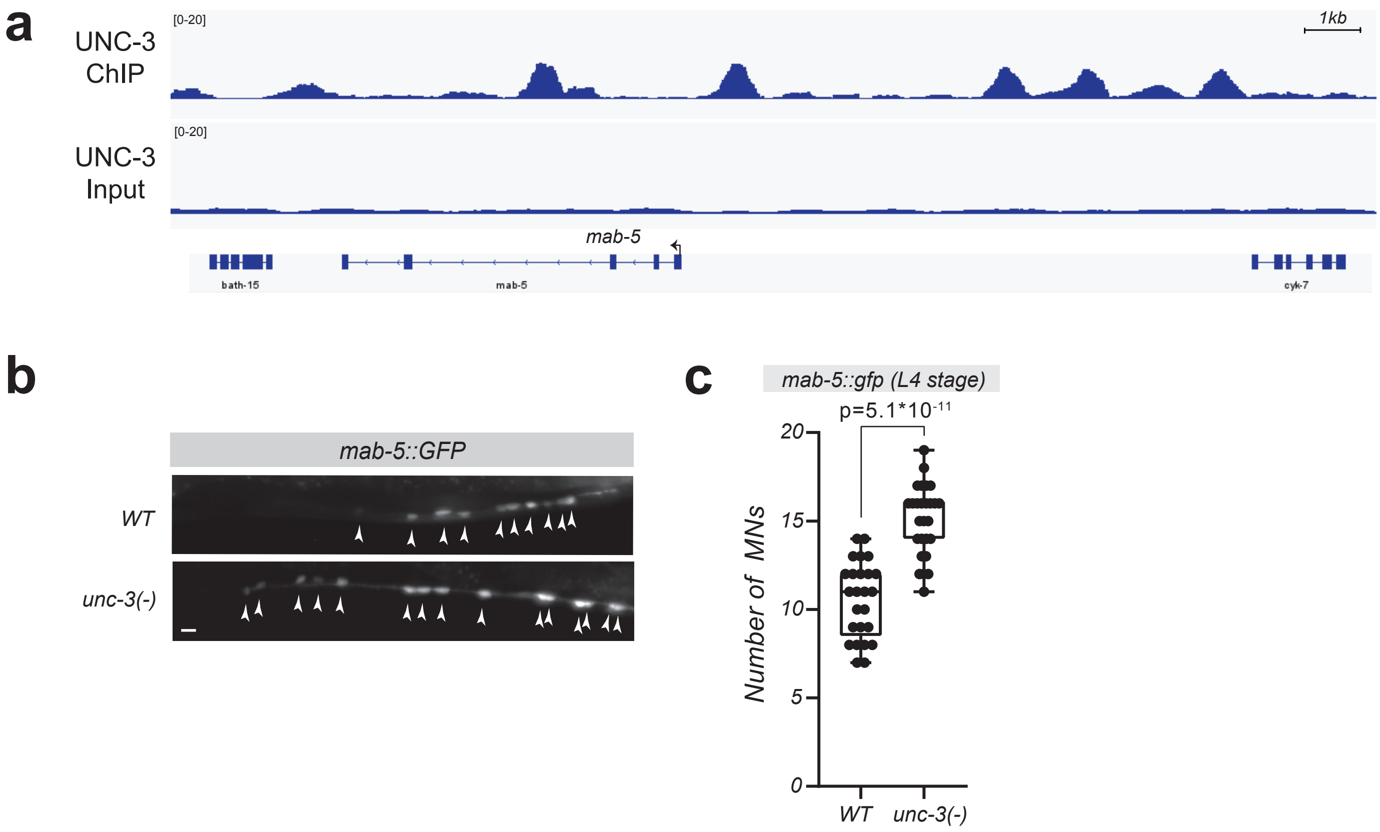

Supplementary Figure 6. UNC-3 represses mab-5 expression.

a. Gene locus of *mab-5* with ChIP-seq tracks for UNC-3. **b**. Representative images of a *mab-5* transcriptional *gfp* reporter in posterior motor neurons of WT and *unc-3(n3435)* mutant animals at L4. **c**. Quantification of *mab-5::gfp* expression in panel **b.** Box and whisker plots were used with presentation of all data points. Box boundaries indicate the 25th and 75th percentile. The limits indicate minima and maxima values, whereas centre values (mean) are highlighted in red. Unpaired t-test (two-sided) with Welch's correction was performed and p-values were annotated. N = 25-27 animals. Source data are provided as a Source data file. Scale bar: 5 μm.

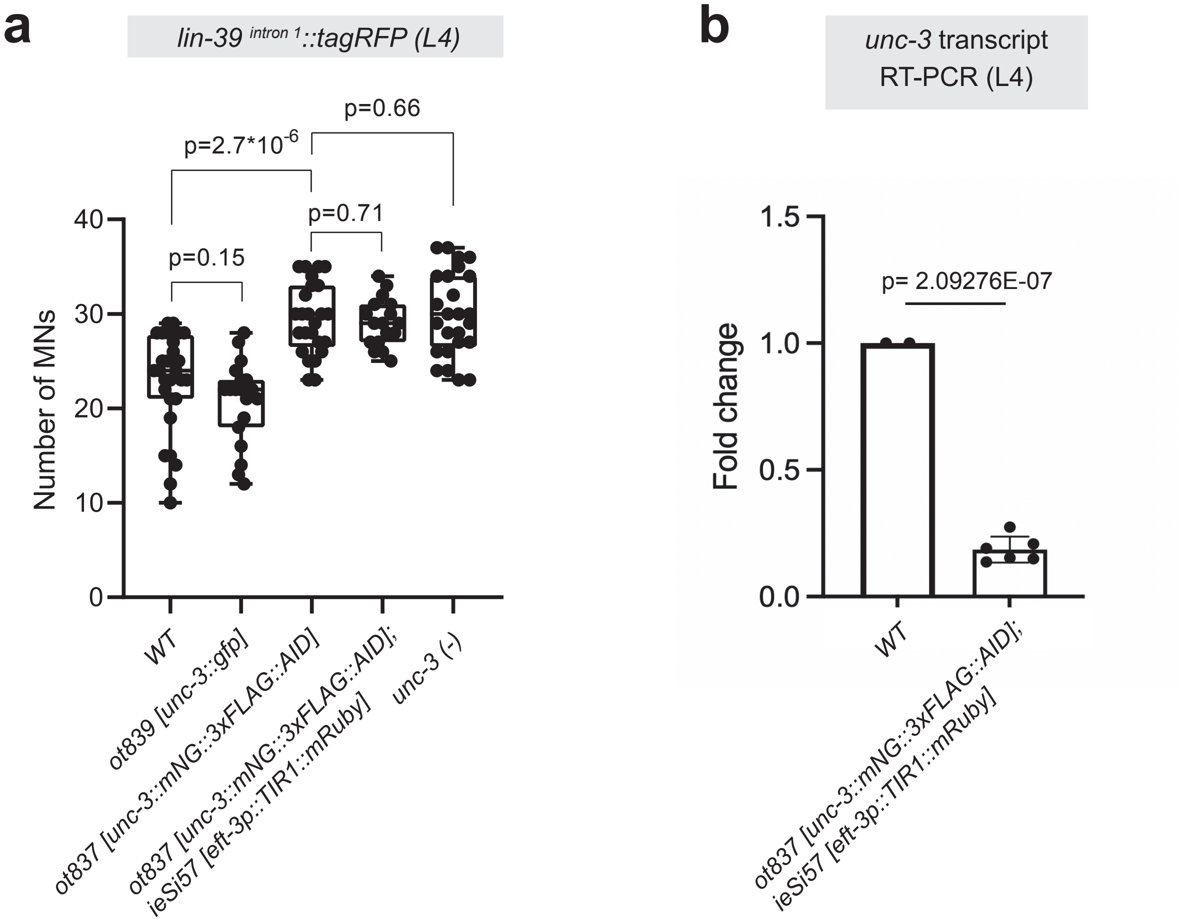

Supplementary Figure 7. The expression of *lin-39* is increased in animals carrying a hypomorphic *unc-3* allele. **a.** Quantification at the L4 stage of the number of MNs expressing *lin-39 intron1::tagRFP* in WT and animals carrying various *unc-3* alleles *(ot839 [unc-3::gfp], ot837 [unc-3::mNG::3xFLAG::AID], ot837 [unc-3::mNG::3xFLAG::AID]; ieSi57 [eft-3p::TIR1::mRuby]*, and *unc-3(n3435)). ot839* is an endogenous gfp reporter allele for *unc-3*, whereas animals carrying the hypomorphic *ot837* allele do show an increase in the number of motor neurons expressing the tagRFP reporter independently of the presence of TIR1 (*ieSi57* transgene). n = 17-28 animals. Box boundaries indicate the 25th and 75th percentile. The limits indicate minima and maxima values, whereas centre values (mean) are highlighted in red. **b.** RT-PCR on whole worm lysates was used to measure *unc-3* mRNA levels at synchronized animals (L4 stage). Compared to wild-type (N2 strain) animals, the levels of *unc-3* transcripts are reduced in *ot837 [unc-3::mNG::3xFLAG::AID]; ieSi57 [eft-3p::TIR1::mRuby]* animals. Data are presented as mean values with error bars indicating standard deviation (S.D.). Unpaired t-test (two-sided) with Welch's correction was performed and p-values were annotated. The experiment was repeated twice. Source data are provided as a Source data file.

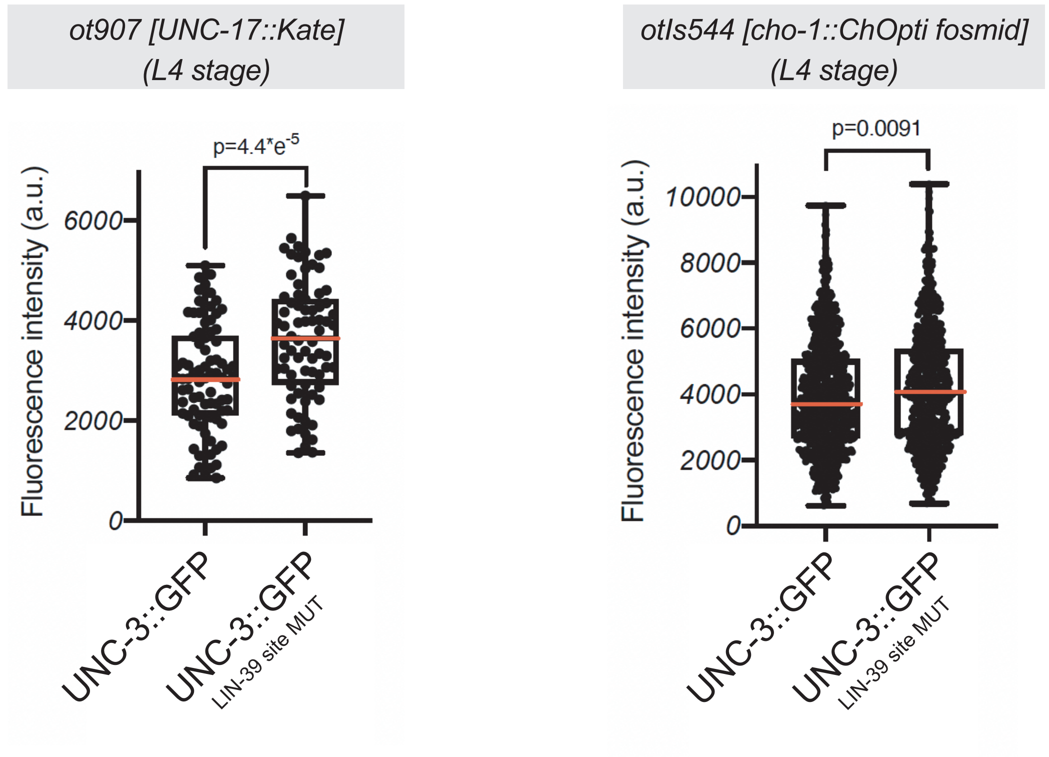

Supplementary Figure 8. Expression of cholinergic identity genes is increased in animals carrying a hypomorphic *unc-3* allele.

The expression levels of cholinergic identity gene markers (*unc-17/VAChT, cho-1/ChT*) are increased (quantification of the levels of fluorescent intensity) in motor neurons of animals carrying the *UNC-3::GFP LIN-39 site MUT* allele (shown in **Fig. 5a**) compared to animals carrying the endogenous *unc-3* reporter allele *ot839 [unc-3::gfp].* Animals were imaged at the L4 stage. Box boundaries indicate the 25th and 75th percentile. The limits indicate minima and maxima values, whereas centre values (mean) are highlighted in red. n = 77 neurons for UNC-17::mKate; n = 608 neurons for *cho-1::mChOpti*. Unpaired t-test (two-sided) with Welch's correction was performed and p-values were annotated.
