# Supplementary material for: Maintenance of neurotransmitter identity by Hox proteins through a homeostatic mechanism
Source: Nat Commun. 2022 Oct 15;13:6097. doi: 10.1038/s41467-022-33781-0 (PMC9569373; doi:10.1038/s41467-022-33781-0)
Supplement: Supplementary file 3 — Description of Additional Supplementary Files [file 41467_2022_33781_MOESM3_ESM.pdf]

Description of Additional Supplementary Files

File Name: Supplementary Data 1
Description: Summary of C. elegans strains used in this study
