# Supplementary material for: Maintenance of neurotransmitter identity by Hox proteins through a homeostatic mechanism
Source: Nat Commun. 2022 Oct 15;13:6097. doi: 10.1038/s41467-022-33781-0 (PMC9569373; doi:10.1038/s41467-022-33781-0)
Supplement: Supplementary file 5 — Reporting Summary [file 41467_2022_33781_MOESM5_ESM.pdf]

nature portfolio

Corresponding author(s): Paschalis Kratsios

Last updated by author(s): 09/22/2022

Reporting Summary

Nature Portfolio wishes to improve the reproducibility of the work that we publish. This form provides structure for consistency and transparency in reporting. For further information on Nature Portfolio policies, see our Editorial Policies and the Editorial Policy Checklist.

Statistics

For all statistical analyses, confirm that the following items are present in the figure legend, table legend, main text, or Methods section.

n/a	Confirmed	
☐	☒	The exact sample size (*n*) for each experimental group/condition, given as a discrete number and unit of measurement
☐	☒	A statement on whether measurements were taken from distinct samples or whether the same sample was measured repeatedly
☐	☒	The statistical test(s) used AND whether they are one- or two-sided<br>*Only common tests should be described solely by name; describe more complex techniques in the Methods section.*
☒	☐	A description of all covariates tested
☒	☐	A description of any assumptions or corrections, such as tests of normality and adjustment for multiple comparisons
☐	☒	A full description of the statistical parameters including central tendency (e.g. means) or other basic estimates (e.g. regression coefficient) AND variation (e.g. standard deviation) or associated estimates of uncertainty (e.g. confidence intervals)
☐	☒	For null hypothesis testing, the test statistic (e.g. *F*, *t*, *r*) with confidence intervals, effect sizes, degrees of freedom and *P* value noted<br>*Give P values as exact values whenever suitable.*
☒	☐	For Bayesian analysis, information on the choice of priors and Markov chain Monte Carlo settings
☒	☐	For hierarchical and complex designs, identification of the appropriate level for tests and full reporting of outcomes
☒	☐	Estimates of effect sizes (e.g. Cohen's *d*, Pearson's *r*), indicating how they were calculated

Our web collection on statistics for biologists contains articles on many of the points above.

Software and code

Policy information about availability of computer code

Data collection	Imaging data for fluorescence reporters and single molecule RNA-FISH experiments were acquired using Axio Imager Z2 (Zeiss) and ZEN software (Zeiss, Version 2.3.69.1000, Blue edition). Image processing was performed using Image J software (version 2.0.0-rc-59/1.51k). To predict transcription factor binding sites, we used (1) the online MatInspector software tool from Genomatix(https://www.genomatix.de/online_help/help_matinspector/matinspector_help.html), and (2) FIMO (Find Individual Motif Occurrences), which is one of the motif-based sequence analysis tools of MEME (Multiple Expectation maximization for Motif Elicitation) bioinformatics suite (http://meme-suite.org/).
Data analysis	Statistical analysis was performed by GraphPad Prism software (version 9.2.0).

For manuscripts utilizing custom algorithms or software that are central to the research but not yet described in published literature, software must be made available to editors and reviewers. We strongly encourage code deposition in a community repository (e.g. GitHub). See the Nature Portfolio guidelines for submitting code & software for further information.

Data

Policy information about availability of data

All manuscripts must include a data availability statement. This statement should provide the following information, where applicable:

- Accession codes, unique identifiers, or web links for publicly available datasets
- A description of any restrictions on data availability
- For clinical datasets or third party data, please ensure that the statement adheres to our policy

The data supporting the findings of this study are included in the figures and supporting files. Source data are provided with this paper. The ChIP-Seq data for UNC-3, LIN-39 and MAB-5 used in this study are available in the NCBI Gene Expression Omnibus (GEO) database under accession codes:
GSE143165 [https://www.ncbi.nlm.nih.gov/geo/query/acc.cgi?acc=GSE143165]
GSE25785 [https://www.ncbi.nlm.nih.gov/geo/query/acc.cgi?acc=GSE25785]
GSE15625 [https://www.ncbi.nlm.nih.gov/geo/query/acc.cgi?acc=GSE15625].

Human research participants

Policy information about studies involving human research participants and Sex and Gender in Research.

Reporting on sex and gender	Not applicable
Population characteristics	Not applicable.
Recruitment	Not applicable.
Ethics oversight	Not applicable.

Note that full information on the approval of the study protocol must also be provided in the manuscript.

Field-specific reporting

Please select the one below that is the best fit for your research. If you are not sure, read the appropriate sections before making your selection.

☒ Life sciences ☐ Behavioural & social sciences ☐ Ecological, evolutionary & environmental sciences

For a reference copy of the document with all sections, see nature.com/documents/nr-reporting-summary-flat.pdf

Life sciences study design

All studies must disclose on these points even when the disclosure is negative.

Sample size	We determined the sample size based on similar experiments described previously in the literature (PMIDs: 25913400, 28056346, 31902393). Based on these studies, the sample size used in this study is sufficient to obtain statistical significance.
Data exclusions	No data were excluded from this study.
Replication	All attempts at replication were successful. Experiments were repeated twice.
Randomization	C. elegans animals with the same genotype were maintained on the same agar plate. For the experiments, animals from multiple genotypes (and thus plates) were used. Animals at a specific developmental stage (see figure legends) were chosen randomly from a population of worms with the same genotype.
Blinding	Cell number and fluorescence intensity quantifications were performed by investigators not blinded to group allocation. Blinding was not possible because the results are quantitative and do not require subjective interpretation.

Reporting for specific materials, systems and methods

We require information from authors about some types of materials, experimental systems and methods used in many studies. Here, indicate whether each material, system or method listed is relevant to your study. If you are not sure if a list item applies to your research, read the appropriate section before selecting a response.

Materials & experimental systems

n/a	Involved in the study	
☒	☐	Antibodies
☒	☐	Eukaryotic cell lines
☒	☐	Palaeontology and archaeology
☐	☒	Animals and other organisms
☒	☐	Clinical data
☒	☐	Dual use research of concern

Methods

n/a	Involved in the study	
☒	☐	ChIP-seq
☒	☐	Flow cytometry
☒	☐	MRI-based neuroimaging

Animals and other research organisms

Policy information about studies involving animals; ARRIVE guidelines recommended for reporting animal research, and Sex and Gender in Research

Laboratory animals	The hermaphrodite Caenorhabditis elegans was used in this study.For the experiments, larval stage 4 (L4) and day 1 adult animals were used.Supplementary file 1 contains all the C. elegans strains used in this study.
Wild animals	No wild animals were used in this study.
Reporting on sex	Hermaphrodite C. elegans animals were used in this study.
Field-collected samples	No field collected samples were used in this study.
Ethics oversight	The study did not require ethics oversight because it employs a very simple invertebrate nematode (C. elegans).

Note that full information on the approval of the study protocol must also be provided in the manuscript.
